# Improvement of Nutrient Uptake, Yield of Black Sesame (*Sesamum indicum* L.), and Alluvial Soil Fertility in Dyke by Spent Rice Straw from Mushroom Cultivation as Biofertilizer Containing Potent Strains of *Rhodopseudomonas palustris*

**DOI:** 10.1155/2023/1954632

**Published:** 2023-04-24

**Authors:** Nguyen Quoc Khuong, Le Vinh Thuc, Cao Tien Giang, Ly Ngoc Thanh Xuan, Le Thi My Thu, Akagi Isao, Sakagami Jun-Ichi

**Affiliations:** ^1^Faculty of Crop Science, College of Agriculture, Can Tho University, Can Tho 94000, Vietnam; ^2^Experimental and Practical Area, An Giang University, Long Xuyen, An Giang 90000, Vietnam; ^3^Vietnam National University, Ho Chi Minh City, Vietnam; ^4^Faculty of Agriculture, Kagoshima University, Kagoashima 890-8580, Japan

## Abstract

The aim of the current research was to evaluate the effects of members of purple nonsulfur bacteria (PNSB), *Rhodopseudomonas palustris* strains of VNW02, TLS06, VNW64, and VNS89, mixed with spent rice straw (SRS) from mushroom cultivation as a carrier on promoting sesame growth and yield, and ameliorating the alluvial soil (AS) fertility in dykes. A 4 × 3 factorial experiment consisting of different levels of the solid PNSB biofertilizer mixture at 0, 3, 4, and 5 t·ha^−1^ (0, 1.81 × 10^8^, 2.24 × 10^8^, and 2.68 × 10^8^ cells pot^−1^, respectively), and nitrogen (N) and phosphorus (P) inorganic fertilizer rates (100, 75, and 50 kg·N·ha^−1^; 60, 45, and 30 kg P_2_O_5_·ha^−1^, respectively) was performed in pots with the sesame variety of ADB1 in the dyked AS. The solid PNSB biofertilizer mixture at at least 3 t·ha^−1^ significantly enhanced the sesame seed yield by providing higher macronutrients for plants by increasing available N and soluble P concentrations in the soil. The solid PNSB biofertilizer mixture in addition to 75% of the recommended N and P fertilizers produced an equivalent yield in comparison to the utilized 100% of N and P inorganic fertilizers. The solid PNSB biofertilizer mixture in the SRS from the mushroom production reduced at least 25% of N and P chemical fertilizers for gaining the maximal seed yield and enriched soil characteristics for the sustainable black sesame cultivation in the dyked AS.

## 1. Introduction

Sesame is considered as a healthy and popular food in Asian countries due to its high oil content, delicious nutty aroma, and flavor [1, 2]. It contains rich nutrients, so it can be used in edible products and for industrial and pharmaceutical aspects [3, 4], while 70% of the global sesame seeds are used to generate oil and meal. The world's sesame seed consumption was 6,559.0 million USD in 2018, and it will be 7,244.9 million USD by 2024 [5]. In Vietnam, black sesame is grown popularly in the Mekong Delta [6], with an area of 29,059 ha and a mean domestic yield of 0.7313 t·ha^−1^ in 2018 [7].

Alluvial soil (AS) is a soil that is deposited by the surface water and can be found along rivers and their banks, in floodplains and deltas. This leads to high nutrient concentrations within the AS; thereby, the soil in An Giang province has been intensively used for crops that lack available nutrients from their indigenous soil [8]. However, because this area is the headwater in the Mekong Delta, the government has established dykes to prevent floods so as to cultivate plants during flooded seasons. As a result, the AS in dykes possessed low fertility for cultivation, especially for black sesame [9].

The use of fertilizers for sesame is a notorious concern in most aspects in developing countries [10], particularly in Vietnam. Therefore, it is vital to find alternative sources to replace chemical fertilizers that can cause adverse impacts on the soil and environment. By recycling agricultural wastes, rice straw (RS) has been used to produce composts as a source of both plant nutrients and microorganisms to improve crop yields and the soil fertility. In addition, the RS has been used for mushroom cultivation; after the mushrooms are harvested, RS turns into spent rice straw (SRS) which can be used as a compost. However, the SRS is a poor soil improver due to its low nutrient value and is poorly decomposed [11]. The spent rice straw has become an organic waste from mushroom production; therefore, a way should be explored to increase its value in agriculture. Organic fertilizers have been proved for their ability to promote sesame's growth parameters including chlorophyll content, plant heights, number of seeds per capsule, number of capsules per plant, and 1,000-seed weights and seed yields [12, 13]. For instance, the application of organic fertilizers and biofertilizers was able to improve the sesame yield, soil fertility, and seed oil quality with higher contents of oleic, stearic, and palmitic acids, but lower contents of linolenic and linoleic acids [14]. Interestingly, the use of plant growth promoting rhizobacteria (PGPR) processing nitrogen (N) fixing ability and phosphorous (P) solubilization as a biofertilizer could reduce the chemical fertilizer input for sesame [15–17] and enhance its N and P uptake [18, 19].

Purple nonsulfur bacteria (PNSB) are N-fixers with ability to grow in different growth modes as a photoautotroph which can make use of light and carbon dioxide in the environment to produce its own energy via the process of photosynthesis, photoheterotrophs, chemoheterotrophs, and chemoautotrophs depending on the environmental condition [20–22]. This ability allows the PNSB to survive and perform their functions in various habitats such as wetland and upland soils. Successful applications of the PNSB *Rhodopseudomonas* spp. as biofertilizers in paddy fields have been reported to improve adverse effects of soil environments such as acidity, salinity, and toxicity [23–29] to enhance rice growth at different stages [25–28, 30–33] and also rice productivity [29, 33]. Additionally, the PNSB promoted the growth of various crops such as sweet herb *Stevia rebaudiana* [34], cucumber seedling [35, 36], and pineapple [37]. Regarding previous studies, it has been suggested that the PNSB can be applied in both wetland and cultivated soils for annual crops; thereby, it would be worth to explore their roles for promoting growth and yield of sesame, particularly in dyked AS.

Interestingly, the biofertilizers of acidic-resistant*Rhodopseudomonas* strains, particularly *R. palustris* VNW02, TLS06, VNW64, and VNS89 strains which were isolated from various acid sulfate soils [38] can act as plant growth promoters and biofertilizers by reducing Al^3+^ and Fe^2+^ [23]. Fortunately, the bacteria prefer to grow in the neutral condition, so it would be possible for them to perform their benefits in the AS for promoting sesame growth. In addition to fixing N, the PNSB can solubilize P [20, 33] and release metabolites such as siderophores, indole-3-acetic acid (IAA), and 5-aminolevulinic acid (ALA) [20] to support plants to overcome adverse conditions and low nutrient inputs. Hence, this research aims to evaluate the efficiency of promising strains of *R. palustris* in SRS from mushroom cultivation as a carrier to support sesame growth and yield, along with improving the AS fertility in dykes, for the possibility to achieve an alternative approach to reduce chemical fertilizers for a sustainable sesame cultivation.

## 2. Materials and Methods

### 2.1. Materials

#### 2.1.1. The Alluvial Soil Used

The soil used in the current study was collected at 0–20 cm depth from a previously sesame cultivated soil in Chau Phu district, An Giang province, Viet Nam. Results of the initial soil physiochemical parameters, which were determined following the method in Section 2.2.3, are given in Table 1.

#### 2.1.2. The Bacteria Used

The four promising strains of *R. palustris*, VNW02, TLS06, VNW64, and VNS89, were used as a mixed biofertilizer in this study, and their accession numbers from the National Center for Biotechnology Information (NCBI) were KY624605, KY624609, KY624606, and KY624607, respectively [38].

#### 2.1.3. Spent Rice Straw

The SRS originating from mushroom cultivation was used as a carrier for the mixed biofertilizer.

#### 2.1.4. Sesame Variety

The black sesame variety ADB1, which is commonly planted in the Mekong Delta, was collected and kept in the Crop Science Faculty, College of Agriculture, Can Tho University.

#### 2.1.5. Pot

Each plastic pot, whose size was 25 and 21 cm in diameter at the top and bottom and 21 cm in height, was used and had seven holes at the bottom.

#### 2.1.6. Chemical Fertilizers

The actual chemical fertilizers used were urea for the N application (46% N), superphosphate fertilizer for the P application (16% P_2_O_5_), and potassium chloride for the K application (60% KCl).

### 2.2. Methods

#### 2.2.1. Preparation of PNSB Inoculants

*(1) PNSB Preparation*. The four PNSB strains were activated in the basic isolation medium (BIM) broth for 48 h under microaerobic light conditions. After that, the optical density of each culture was adjusted to 1.2 at the wavelength of 660 nm by diluting in sterile distilled water, with the final density of 10^9^ cells·mL^−1^. Each cell suspension was ready to be used as an inoculant.

*(2) Sesame Seed Preparation and Bacterization*. The black sesame seeds were first sanitized by submergence in 70% ethanol for 3 min and 1% sodium hypochlorite for 10 min, and then washed twice with sterile distilled water. The black sesame seeds were incubated for 24 h under dark conditions for germination. Subsequently, for the seed bacterization, 1,000 sesame seeds were mixed well with a 10 mL bacterial cell suspension of 10^9^ cells·mL^−1^ (2.5 mL for each strain) for 1 h in a reciprocal shaker at 60 rpm. After 2 h of mixing, the seeds were taken out and dried under the laminar air flow before planting. The control seeds were similarly prepared, but in sterile distilled water instead of the bacterial suspension. Five seeds were grown in a pot, so the density of the mixed 4 PNSB was roughly 0.5 × 10^8^ cells pot^−1^.

*(3) Preparation of the Mixed PNSB Biofertilizer in a Solid Form*. The SRS from mushroom cultivation was sterilized at 121°C for 30 min and was used as a carrier for the PNSB biofertilizer based on its low nutrient content and poor decomposition. Then, 10 mL of 4 PNSB inoculants (2.5 × 4 each) was added to 100 g of the carrier and incubated for one week before use. This product was named as the solid mixed PNSB biofertilizer containing roughly 10^8^ cells g^−1^ and is called as solid PNSB biofertilizer in short.

*(4) Application of the Solid Mixed PNSB Biofertilizer*. A 50% of the designated weight of the solid PNSB biofertilizer was applied on the soil surface at 0 and 20 days after sowing. It was put near sesame roots with designated levels. The solid PNSB biofertilizer consisted of 2.37% N, 1.76% P, 1.20% K, and C/N ratio (20.0); the PNSB density was 0.87 × 10^7^ cells·g^−1^. Thus, the total PNSB density in each pot was 1.81 × 10^8^ cells pot^−1^ that corresponded to 3 t·ha^−1^, 2.24 × 10^8^ cells pot^−1^ for 4 t·ha^−1^, and 2.68 × 10^8^ cells pot^−1^ for 5 t·ha^−1^. The methods were described by Sparks et al. [39]. In detail, the total N, P, K, and C contents were measured by the Kjeldahl, inductively coupled plasma-optical emission spectroscopy (ICP-OES), and ash methods, respectively.

#### 2.2.2. Soil and Fertilizer Preparation for Planting

*(1) Soil Preparation*. RS residues were removed from the used soil before mixing to avoid its effects on the soil fertility and drying in the ambient atmosphere. Then, 10 kg of soil in each pot was moistened with roughly 1 L pot^−1^ of tap water for 2 days before sowing.

*(2) Chemical Fertilizer Formula*. N, P, and K fertilizers were used as the recommendation formula of 100N-60P_2_O_5_-30K_2_O. Each treatment was applied as a designated volume. The fertilization was divided into 4 times. For the first time, 100% phosphorus was applied as a base. In the second time, 10 days after sowing (DAS), 30% N and 50% K were applied. In the third time, 40% N was applied at 20 DAS. Finally, 30% N and 50% K were applied [6].

*(3) Sesame Planting*. Each pot contained 10 kg of dry soil with five plants (one plant in the center and four plants at four corners).

#### 2.2.3. Analyzed Methods of Soil Property

*(1) Initial and Harvest Soil Property*. The methods for the soil analysis are described as follows: Soil chemical parameters in Table 1 were analyzed using methods described by Sparks et al. [39]. Briefly, for the pH and electrical conductivity (EC) measurements, soil samples were separately extracted using 1 M KCl (pH_KCl_) or deionized water (pH_H2O_) at a ratio of 1 : 5 for soil : solvent. The total iron was determined by using the ICP-OES. The Al_exchangeable_ concentration was extracted by 1 M KCl, and the spectrophotometric method was employed for the aluminum ion quantization from the soil supernatant by the fluorometric analysis of the Al^3+^ content with 8-hydroxyquinoline and butyl acetate. The Fe_active_ concentration in soil samples was extracted by diethylenetriaminepentaacetic acid (DTPA). The digested solution was used for the amorphous iron oxide analysis, which was analyzed by ammonium oxalate acid in the dark and detected by the modified Tamm's reagent. The ferrozine method was described for determination of the Fe^2+^ concentration via the 1,10-phenanthroline reagent using a visible spectrophotometer at the wavelength of 562 nm. The available P was determined by the Bray II methods, and fractions of inorganic P were extracted by 0.5 M NH_4_F, 0.1 M NaOH, and 0.25 M H_2_SO_4_ for aluminum-phosphorus (Al-P), iron-phosphorus (Fe-P), and calcium-phosphorus (Ca-P), respectively. The total N was quantified by the regular Kjeldahl method via destruction of organic N prior to the inorganic N determination. The NH_4_^+^−N concentration was distillated by a titrimetric procedure. The dichromate oxidation procedure by the thermal conductivity technique of sulfuric acid was utilized for converting to the inorganic carbon from organic carbon compounds prior to titrate with ferrous sulfate heptahydrate for the total carbon determination. The cation exchange capacity (CEC): A compulsive exchange method has been recommended for the determination of CEC in the soil. This extracted solution was also used for the analysis of potassium, sodium, calcium, and magnesium by an atomic absorption spectrophotometry.

*(2) PNSB Population*. The density of the PNSB from the harvest soil was enumerated by the most probable number technique as described by Harada et al. [40] and modified by Kantachote et al. [29].

#### 2.2.4. Experimental Design

A two factorial experiment on sesame cultivation was designed in completely randomized blocks for adding the solid PNSB biofertilizer with four levels of 0, 3, 4, and 5 t·ha^−1^ and the chemical fertilizer with three levels of 50, 75, and 100% of N and P of the recommendation formula of chemical fertilizer (RC). Therefore, the experiment consisted of 12 sets as follows: 3 t·ha^−1^ of the biofertilizer with 50, 75, and 100% of N and P; 4 t·ha^−1^ of the biofertilizer with 50, 75, and 100% of N and P; and 5 t·ha^−1^ of biofertilizer with 50, 75, and 100% of N and P, with 4 replications. The experiment was carried out in the greenhouse for 85 days.

#### 2.2.5. Measurements of Agronomic Parameters

*(1) Sesame Growth Parameters*. The plant height (cm) was measured from the soil surface to the highest point of the plants. The number of leaves was counted as the total number of leaves on the main stem. The length of leaves was determined from the leaf collar to the leaf tip. The width of leaves was determined at the largest size of a leaf. The number of branches was counted from the bottom to the top, with the length of branch at least 3 cm. The number of capsules per plant was counted at harvest. The length of capsule was determined between two tips of a capsule. The dry biomass: Components of stem-leaf, shell, and seed were dried at 70°C for 72 h and the dry biomass was weighed. All parameters were measured according to Fazeli Kakhki et al. [18].

*(2) Biochemical Parameters*. The leaf chlorophyll content was determined by Moran [41]. The 10^th^ leaf from the soil surface of 5 plants was collected after 30 days of sowing. A total of 1 cm^2^ of each leaf was added to each Eppendorf containing 3 mL·N,N-dimethylformamide for 24 h in an incubator at 25°C. The solution was determined by a spectrophotometer at the wavelengths of 664 and 647 nm for detecting chlorophyll *a* and *b*, respectively. Afterwards, chlorophyll *a* and *b* and total chlorophyll contents (*C*_*a*_, *C*_*b*_, and *C*_*t*_) were calculated as follows:

(1) $$\begin{array}{c} A_{664}=83.9\;C_{a}+10.8\;C_{b},A_{647}=20.2\;C_{a}+45.6\;C_{b,}\;C_{a}=12.64\;A_{664}\;–\;2.99\;A_{647},C_{b}=-5.6\;A_{664}+23.26\;A_{647}\;C\left(t\right)=7.04\;A\left(664\right)+20.27\;A_{647}\text{.} \end{array}$$

The Soil Plant Analysis Development (SPAD) index was directly detected on the 12^th^ leaf from the soil surface by SPAD-502 Plus according to the guide of the Konica Minolta manufacturer.

*(3) Agronomic Traits*. Sesame yield components and yields were determined on the capsule length, capsule diameter, number of capsules plant^−1^, number of filled capsules plant^−1^, percentage of capsules plant^−1^, number of seeds capsule^−1^, number of filled seeds capsule^−1^, percentage of filled seeds capsules^−1^, and a-thousand-seed oven-dry weight.

#### 2.2.6. Analyzed Methods of Plant Property

*(1) Analysis of N_total_, P_total_, and K_total_ Concentrations in Sesame at Harvest*. The stem-leaf, shell, and seed samples of each set were collected and dried at 70°C for 72 h. Then, they were ground to pass through a 0.5 mm sieve for the total analysis of all parameters. The N_total_, P_total_, and K_total_ in stem-leaf, shell, and seed were investigated. The total N, P, and K concentrations were determined by following the methods of Houba et al. [42]. The N_total_ was determined by the regular Kjeldahl method, the P_total_ concentration was determined by colorimetric procedure at the wavelength of 880 nm, and the K_total_ content was determined by using the ICP-OES.

*(2) Determination of N_total_, P_total_, and K_total_ Accumulation in Sesame at Harvest*. The amount of N, P, and K uptake was calculated from stem-leaf, shell, and seed biomass multiplied by each concentration of each component. The total uptake was calculated from all parts.

### 2.3. Statistical Analysis

The data shown in this paper are mean values of the four replications. The data were subjected to the two-way analysis of variance (ANOVA) using the SPSS software package version 13.0. The significant differences were assessed by Duncan's post hoc tests at *P* < 0.05.

## 3. Results

### 3.1. Effects of the Solid PNSB Biofertilizer on the Growth of Sesame

The application of the solid PNSB biofertilizer significantly improved the plant height, number of leaves, and number of branches compared with the control (without the PNSB biofertilizer) (Table 2). The plant height (65.7–68.3 cm) while being supplemented from 3 to 5 t·ha^−1^ of the solid PNSB biofertilizer was significantly higher than the control (63.4 cm). This led to a much higher number of leaves as well. However, no improvement in length and width of leaves by the solid PNSB biofertilizer was observed. For the chemical fertilizer, the reduction in amounts of N and P significantly decreased the plant height, number of leaves, and width of leaves, i.e., the plant height of the treatment applied with 50–75% N and P of the RC achieved 63.5–66.2 cm, and it was lower than the 100% N treatment, 68.6 cm (Table 2).

The concentration of the chlorophyll*a* and the total chlorophyll were higher in treatments applied with 75–100% N and P of the RC as compared with the one with 50% N and P of RC, with 10.7–10.9 and 8.78 *µ*g·Chl·mL^−1^ and 13.7–13.9 and 11.2 *µ*g·Chl·mL^−1^. The SPAD value also showed the same trend (Table 2).

### 3.2. Effects of the Solid PNSB Biofertilizer on the Nutrient Uptake

Both factors (the biofertilizer and the chemical fertilizer) produced a positive effect on the N concentration, with higher *N* values in plant parts of stem-leaf, shell, and seed (0.16–0.19, 0.16–0.21, and 0.92–0.94% DW) in the treatments applied with the solid PNSB biofertilizer, compared with 0.14, 0.15, and 0.86% DW showed by the control (no added PNSB biofertilizer). The P concentrations showed a similar trend, with an exception for P content in seed. The K concentration was in the same pattern with the N concentration, its value with and without application found in stem-leaf (4.84–4.98 and 4.24% DW), shell (1.74–2.44 and 1.57% DW), and seed (0.15-0.16 and 0.12% DW). Increasing of N and P fertilizers input also enhanced N and P concentrations in seeds to 0.86, 0.90, and 0.96% N and 0.74, 0.76, and 0.76% P as in the order of 50, 75, and 100% N and P of the RC (Table 3).

The use of the solid PNSB biofertilizer at different levels significantly increased the plant biomass in stem-leaf (8.68–9.10 g·pot^−1^), shell (6.62–6.95 g·pot^−1^), and seed (11.4–13.3 g·pot^−1^) compared with the no application (control) with 7.67, 5.75, and 9.4 g·pot^−1^, respectively (Table 3). The highest biomass was obtained at 100% N and P of the RC, with 10.2 g·pot^−1^ in stem-leaf, 7.71 g·pot^−1^ in shell, and 14.7 g·pot^−1^ in seed, followed by 75% N and P of the RC with 8.5, 6.28, and 11.1 g·pot^−1^. The lowest biomass was recorded at 50% N and P of the RC with detail values of 7.0, 5.62, and 9.73 g·pot^−1^ in stem-leaf, shell, and seed, respectively (Table 3).

**Figure 1:**
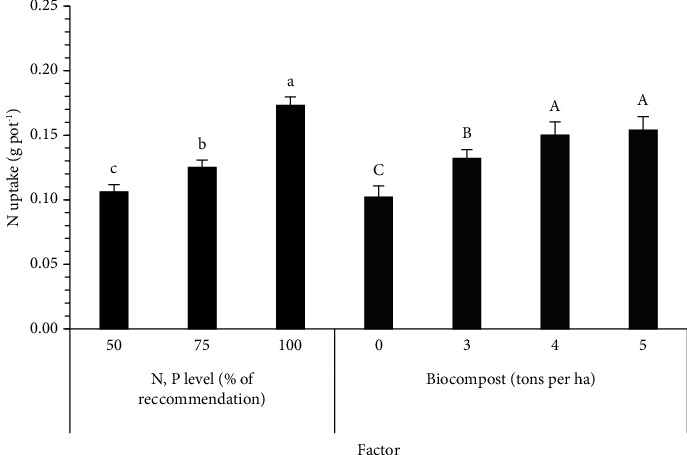
Effects of different levels of mixed *Rhodopseudomonas palustris* strains in spent rice straw and chemical fertilizer on N uptake of sesame grown in dyked alluvial soil under greenhouse conditions. Different lowercase and uppercase letters indicate significant differences in N, P, and biofertilizer levels.

**Figure 2:**
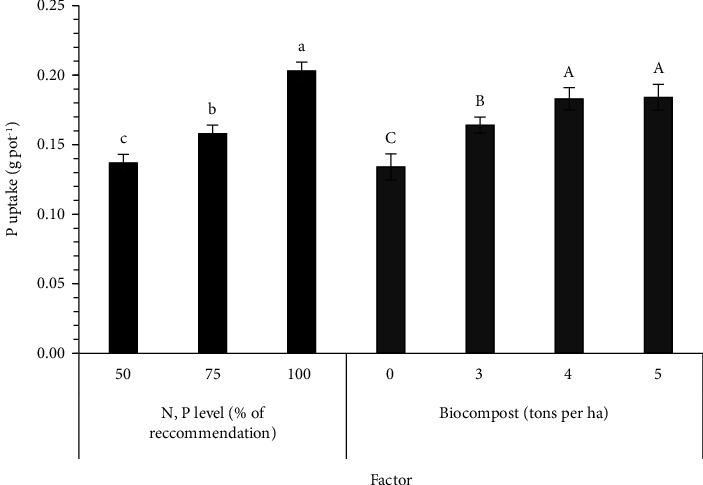
Effects of different levels of mixed *Rhodopseudomonas palustris* strains in spent rice straw and chemical fertilizer on P uptake of sesame grown in dyked alluvial soil under greenhouse conditions. Different lowercase and uppercase letters indicate significant differences in N, P, and biofertilizer levels.

**Figure 3:**
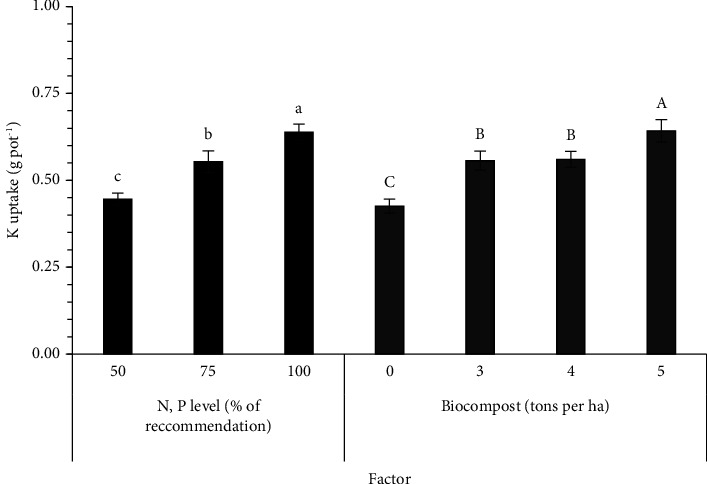
Effects of different levels of mixed *Rhodopseudomonas palustris* strains in spent rice straw and chemical fertilizer on K uptake of sesame grown in dyked alluvial soil under greenhouse conditions. Different lowercase and uppercase letters indicate significant differences in N, P, and biofertilizer levels.

The N, P, and K uptake values in sesame parts are recorded in Table 4. There were significant differences in the N, P, and K uptake between the solid PNSB biofertilizer level and between the N and P fertilizers input level (Table 4). A decrease in adding both factors reduced the total N uptake as follows: 0.173, 0.125, and 0.106 g·pot^−1^ for 100, 75, and 50% N and P of the RC, respectively, while it was 0.154, 0.150, 0.132, and 0.102 g·pot^−1^ for 5, 4, 3, and 0 t·ha^−1^ of the solid PNSB biofertilizer (Figure 1). The total P uptake showed the same trend with 0.203 g·pot^−1^ for 100% N and P of the RC, 0.158 g·pot^−1^ for 75% N and P of the RC, and 0.137 g·pot^−1^ for 50% N and P of the RC and ranged 0.134–0.184 g·pot^−1^ for the different solid PNSB biofertilizer levels (Figure 2). Regarding the total K uptake, the result ranged 0.446–0.693 g·pot^−1^ between the recommended N and P fertilizer levels and 0.426–0.642 g·pot^−1^ among the solid PNSB biofertilizer levels (Figure 3).

### 3.3. Effects of the Solid PNSB Biofertilizer on the Yield Components and Yield of Sesame

The solid PNSB biofertilizer application contributed to a significant increase in yield components including the length of capsule, number of capsules plant^−1^, number of filled capsules plant^−1^, and ratio of filled seeds capsule^−1^, with 2.45–2.56 cm, 15.3–17.1 capsules, 14.8–15.9 capsules, 118.3–123.9 seeds, and 95.0–99.7%, respectively, while in the control, values were correspondingly 2.40 cm, 12.6 capsules, 11.9 capsules, 112.2 seeds, and 94.4%. The decrease of N and P fertilizers resulted in a significant decrease in the diameter of capsule and number of seeds per capsule. However, there was no significant difference between two factors in the weight of 1,000 seeds, with the mean value of 2.45 g. An increase in adding N and P fertilizers significantly improved the seed yield, with the volume of 50, 75, and 100% as in the degree of 10.5 < 12.1 < 15.9 g·pot^−1^. Thus, the supplement of the solid PNSB biofertilizer produced a higher yield than the control, with 12.3–14.5 g·pot^−1^ in comparison with 10.3 g·pot^−1^ (Table 5).

### 3.4. Effects of the Solid PNSB Biofertilizer Mixture on the Soil Fertility

The application of the solid PNSB biofertilizer helped to reduce acidity as pH values of treatments were in a range of 6.28–6.48, which were significantly higher than the control (6.09). It is a similar trend to the potential acidity of pH_KCl_ in the applied PNSB biofertilizer, with higher pH of 5.43–5.73 in comparison with 5.12 in the control. However, no significant difference was found for the chemical fertilizer among N and P levels in pH_H2O_ and pH_KCl_ (Table 6).

The amendment of the solid PNSB biofertilizer significantly enhanced the concentrations of both the total N and total P; however, the chemical fertilizers application did not change their concentrations in the soil. Specifically, the total N and P were determined as 0.213–0.232% N and 0.039–0.041% P in the solid PNSB biofertilizer treatments, while in the control, they were 0.185% N and 0.031% P. Similarly, the solid PNSB biofertilizer application significantly increased the available ammonium, with the concentrations of 0, 3, 4, and 5 t·ha^−1^ as 98.2∼87.8 < 128.2 < 136.5 mg NH_4_^+^ kg^−1^, whilst in the chemical fertilizer, results were recorded as 126.2 mg NH_4_^+^ kg^−1^ for 100% of the RC, 108.3 mg NH_4_^+^ kg^−1^ for 75% of the RC, and 103.7 mg NH_4_^+^ kg^−1^ for 50% of the RC (Table 6).

For the P content in the soil, the amendment of the solid PNSB biofertilizer significantly increased the total P, but the N and P chemical fertilizers application still maintained the same concentration. This led to a significant increase in the soluble P in the soil for the solid PNSB biofertilizer sets. For example, treatments of the solid PNSB biofertilizer possessed 34.9–43.6 mg·P·kg^−1^, but it was only 28.8 mg·P·kg^−1^ in the control. Moreover, the P fractions in application with solid PNSB biofertilizer were less than in without application as in the Al-P (51.8–62.9, 72.9 mg·kg^−1^), Fe-P (316.3–317.4, 329.4 mg·kg^−1^), and Ca-P (123.4–127.1, 146.1 mg·kg^−1^) concentrations, respectively (Table 6).

The organic matter content was significantly different between adding the solid PNSB biofertilizer and no PNSB addition as the control. The former soil was analyzed as 2.19–2.67% C, while the latter soil was detected at 1.29% C. Besides, the exchangeable K content in the treatment with the solid PNSB biofertilizer in the former soil was higher than in the control, with 0.445–0.543 and 0.353 meq 100 g^−1^, respectively (Table 6).

## 4. Discussion

Increasing chemical fertilizers resulted in higher sesame growth and yield (Tables 2 and 5). Nitrogen fertilizer plays a key role in increasing the chlorophyll content; it can be seen that the chlorophyll concentration of 75 and 100% N application was significantly higher than that of the dose of 50% (Table 2). It was also confirmed by SPAD values (Table 2). This can be explained by an increase in yield components comprising of the number of capsules, the percentage of filled capsules per plant, the number of seeds per capsule, the percentage of filled seeds per capsule, and the capsule size (Table 5). These results showed that in the indigenous soil, N and P concentrations did not meet the nutrient requirements for sesame, so sesame growth and yield responded to inorganic N and P fertilizers application. This is the reason why the supplementation of the PNSB biofertilizer at 4–6 t·ha^−1^ significantly increased 30.7–39.0% of ammonium and 21.2–51.4% of soluble P as compared with the control. There are reasons for the rise in sesame yield. Firstly, the N, P, and K contents contained in the mixed PNSB biofertilizer was 2.37% N, 1.76% P, and 1.20% K, and in the SRS, lower values of 1.98% N, 0.76% P, and 0.98% K were recorded. Secondly, the ratio of C/N (19.7) is proper for use as a biocompost in agriculture, while that of this study was 28.7.

The PNSB promoted sesame growth by acting as biofertilizers and plant growth promoters that induced plants by releasing substances such as ALA and IAA into the soil [30]. The role of the PNSB biofertilizer containing *R. palustris* TLS06, VNW02, VNW64, and VNS89 strains has been reported for their impacts on rice growth and productivity in an application as a biofertilizer [25, 27, 33]. Other biofertilizers containing strains of PNSB also play a key function to help plant growth. Specifically, the *R. palustris* PSB06 strain stimulated the rice growth at the seedling stage by root elongation of seeds [43], and the *R. palustris* also increased the fresh biomass of rice seedlings [44] and rice grain yield [29, 30]. Thus, the biofertilizer containing the *R. palustris* TLS06, VNW02, VNW64, and VNS89 strains encouraged sesame growth by inoculating into seeds and also in a form of a solid mixed biofertilizer into the soil. Moreover, the *R. palustris* inoculation might help recovery of the soil community to reduce adverse effects caused by chemical fertilization [34].

The application of the solid PNSB biofertilizer mixture resulted in a remarkable improvement in the soil characteristics with higher concentrations of ammonium and soluble P by the activities of N fixation and P solubilization, respectively. It is obvious that the PNSB strains from the biofertilizer fixed N_2_ from the air, so the treatments of the biofertilizer application obtained higher values than those without PNSB biofertilizer application. Similarly, the PNSB from the solid biofertilizer solubilized the insoluble P fractions in the AS in dykes. Thus, the biofertilizer application treatments produced more nutrients than those without PNSB biofertilizer treatments (Table 6). According to Sakpirom et al. [45, 46], the *R. palustris* TN110 strain possessed N fixing genes such as *nifH*, *vnfG*, and *anfG* under various conditions, so it can release the ammonium for sesame, i.e., the ammonium content in the soil was higher by 30.7–39.0% in the treatments applied with 4-5 t·ha−1 of the mixed biofertilizer than in the control (Table 3). The TLS06 and VNW64 strains have genes for N fixation including *nifH* and *anfG* [47]. These PNSB biofertilizers possessed the functions of N fixing and P solubilizing by releasing acid phosphatase and phytase enzymes [20, 27, 33]. Although the function of K solubilization of those PNSB has not been tested in the current research and in the previous experiments, the exchangeable K was still higher in the PNSB biofertilizer treatments than in the treatment with no applied PNSB biofertilizer. This encouraged that the function should be tested in the future for those PNSB as well. According to Ge and Zhang [36], PNSB strains also produced higher K concentrations due to their ability to solubilize K. It should be noted that the PNSB population (6.14–6.92 MPN g^−1^ DSW) from treatments of the solid biofertilizer was significantly higher than without PNSB biofertilizer treatment, 1.94 MPN·g^−1^ DSW. Besides, the application at 3–5 t·ha^−1^ of the solid PNSB biofertilizer supplied much organic matter from the SRS, resulting in higher contents in comparison with the control (Table 6). This means that the solid PNSB biofertilizer performed a huge improvement in soil quality and soil fertility. This is because the biofertilizer containing *R. palustris* TLS06, VNW02, VNW64, and VNS89 strains can ameliorate soil quality by reducing the toxicities of Al^3+^ and Fe^2+^ concentrations by producing exopolymeric substances and siderophores [20, 23] and enrich the soil fertility by increasing NH_4_^+^ and PO_4_^3−^ in acid sulfate soils [27, 33]. PNSB biofertilizers also have ability to reduce Mn^2+^ toxicity [25] and toxicities of other metals, such as Pb, Hg, and Cd, by exopolymers secreted by the *R. palustris* PI5 strain and *Rhodobacter* sp. [48] or the *Rhodobacter sphaeroides* SC01 strain [49].

This PNSB group in the solid mixed biofertilizer produced more available plant nutrients in the soil, so sesame can absorb, contributing to improving the N, P, and K uptake. The percentage of N, P, and K accumulation significantly increased by 29.4–51.0, 22.4–37.3, and 30.8–50.7%, respectively (Figures 1–3). The solid PNSB biofertilizer application promoted the sesame growth under the low N and P input; thereby, the PNSB biofertilizer produced a big increase in the sesame yield by 40.8% (Table 5). To sum up, the biofertilizer containing the *R. palustris* TLS06, VNW02, VNW64, and VNS89 strains showed a promising application as a biofertilizer to improve soil properties, nutrient uptake, and crop yield. Thus, in the application at 4-5 t·ha^−1^ of the solid biofertilizer containing *R. palustris*, TLS06, VNW02, VNW64, and VNS89 strains plus 75% N and P of chemical fertilizers produced the seed yield of 13.7–13.9 g·pot^−1^, which was equal to that of the 100% N and P of chemical fertilizers. Hence, the solid PNSB biofertilizer contributed to reducing 25% of the chemical fertilizers.

## 5. Conclusion

The application at 3, 4, and 5 t·ha^−1^ of SRS containing the biofertilizer of *Rhodopseudomonas palustris* TLS06, VNW02, VNW64, and VNS89 strains in corporation with the chemical fertilizer significantly improved the growth of sesame based on agronomic parameters compared with the control with no PNSB biofertilizer. There was a remarkable increase in the N, P, and K uptake in sesame according to the application of the biofertilizer. The sesame seed yield increased along with the increase in the solid mixed PNSB biofertilizer levels, with 12.3 g·pot^−1^ at 3 t·ha^−1^, 14.5 at 4 t·ha^−1^, and 14.3 at 5 t·ha^−1^ compared with the control, 10.3 g·pot^−1^. Significant increases in the pH value, ammonium, soluble phosphorus, organic matter, and exchangeable potassium indicated that the fertility of the alluvial soil was improved by the solid mixed PNSB biofertilizer for the sustainable sesame cultivation in dykes.

## Figures and Tables

**Table 1:** Initial soil physicochemical properties of dyked alluvial soil collected from upland field cultivated sesame in Vietnam.

Parameter	Value
pH_H2O_	5.86 ± 0.05
pH_KCl_	4.87 ± 0.06
Electrical conductivity, EC (mS·cm^−1^)	156.7 ± 3.96
Organic matter, OM (%)	1.43 ± 0.24

*Nitrogen concentration*	
Total nitrogen (%)	0.16 ± 0.01
Ammonium ion (mg NH_4_^+^·kg^−1^)	35.0 ± 7.79
Nitrate ion (mg NO_3_^−^·kg^−1^)	22.1 ± 0.89

*Phosphorus concentration*	
Total phosphorus (% P)	0.024 ± 0.001
Available phosphorus (mg·P·kg^−1^)	32.1 ± 0.48

*Fractionation of soil phosphorus (mg kg* ^*−*^ *^1^)*	
Aluminum-phosphorus (Al-P)	63.8 ± 4.57
Iron-phosphorus (Fe-P)	284.3 ± 5.71
Calcium-phosphorus (Ca-P)	216.8 ± 7.12

*Exchangeable cations (meq 100 g* ^*−*^ *^1^)*	
K^+^	0.66 ± 0.09
Na^+^	0.63 ± 0.07
Ca^2+^	0.79 ± 0.12
Mg^2+^	2.42 ± 0.24
Cation exchange capacity (CEC)	10.4 ± 0.83
Soil separate (%) and texture	Silty clay
Sand	5.50 ± 0.13
Silt	46.1 ± 3.10
Clay	48.4 ± 1.81

Values are mean and its standard deviation, *n* = 3.

**Table 2:** Effects of different levels of a mixture of four strains of *Rhodopseudomonas palustris* in spent rice straw and chemical fertilizer on sesame growth in dyked alluvial soil under the greenhouse condition.

Factor	Plant height (cm)	Number of leaves (leaf)	Length of leaf (cm)	Width of leaf (cm)	Number of branches (branch)	Chl a^*β*^(*μ*g·Chl·mL^−1^)	Chl b^*β*^(*μ*g·Chl·mL^−1^)	Chl total^*β*^(*μ*g·Chl·mL^−1^)	SPAD index
Biofertilizer level (A) (t·ha^−1^)									
0	63.4^d^	21.9^b^	9.18	4.59	4.96^c^	10.1	2.57	12.7	41.5
3	67.0^b^	24.2^a^	9.49	4.71	5.79^a^	9.83	2.51	12.3	38.8
4	68.3^a^	24.7^a^	10.0	5.09	5.47^ab^	10.5	2.70	13.2	39.0
5	65.7^c^	24.3^a^	9.17	4.49	5.25^bc^	10.1	3.46	13.5	39.9
Nitrogen and phosphorus fertilizer level (B) (%)									
100	68.6^a^	25.5^a^	10.1	5.22^a^	5.47	10.9^a^	2.78	13.7^a^	41.9^a^
75	66.2^b^	24.0^b^	9.50	4.64^bc^	5.41	10.7^a^	3.18	13.9^a^	41.8^a^
50	63.5^c^	21.8^c^	8.77	4.30^c^	5.23	8.78^b^	2.47	11.2^b^	35.8^b^
Significant differences									
(A)	^*∗*^	^*∗*^	ns	ns	^*∗*^	ns	ns	ns	ns
(B)	^*∗*^	^*∗*^	ns	^*∗*^	ns	^*∗*^	ns	^*∗*^	^*∗*^
(A^*∗*^B)	^*∗*^	ns	ns	ns	ns	ns	ns	ns	ns
C.V. (%)	1.49	4.72	16.3	18.7	10.5	14.6	19.4	15.9	12.8

In a column, the different superscripts indicate significant differences at 5% (*P* < 0.05, ^*∗*^) and without a superscript indicates no significant difference according to Duncan's post hoc test at 5% level. Values are means of four replications. ^*β*^The fresh sample was used.

**Table 3:** Effects of different levels of a mixture of four strains of *Rhodopseudomonas palustris* in spent rice straw and chemical fertilizer on nutrient concentration in parts of sesame grown in dyked alluvial soil under the greenhouse condition.

Factor	Nitrogen concentration			Phosphorus concentration			Potassium concentration			Biomass		
	(% DW)									(g·pot^−1^)		
	Stem and leaf	Shell	Seed	Stem and leaf	Shell	Seed	Stem and leaf	Shell	Seed	Stem and leaf	Shell	Seed
Biofertilizer level (A) (t·ha^−1^)												
0	0.14^c^	0.15^c^	0.86^b^	0.45^b^	0.47^b^	0.76	4.24^b^	1.57^c^	0.12^b^	7.67^b^	5.75^c^	9,4^c^
3	0.17^b^	0.16^c^	0.94^a^	0.52^a^	0.48^b^	0.76	4.84^a^	1.79^b^	0.15^a^	8.68^a^	6.62^b^	11,4^b^
4	0.16^b^	0.19^b^	0.92^a^	0.52^a^	0.55^a^	0.75	4.77^a^	1.74^b^	0.15^a^	8.84^a^	6.95^a^	13,3^a^
5	0.19^a^	0.21^a^	0.92^a^	0.55^a^	0.51^ab^	0.74	4.98^a^	2.44^a^	0.16^a^	9.10^a^	6.82^ab^	13,2^a^
Nitrogen and phosphorus fertilizer level (B) (%)												
100	0.17^a^	0.19^a^	0.96^a^	0.51	0.51	0.76^a^	4.55	1.96^a^	0.15	10.2^a^	7.71^a^	14,7^a^
75	0.15^b^	0.17^b^	0.90^b^	0.51	0.49	0.76^a^	4.85	1.93^a^	0.14	8.5^b^	6.28^b^	11,1^b^
50	0.17^a^	0.16^c^	0.86^b^	0.52	0.51	0.74^b^	4.72	1.77^b^	0.14	7.0^c^	5.62^c^	9,73^c^
Significant differences												
(A)	^*∗*^	^*∗*^	^*∗*^	^*∗*^	^*∗*^	ns	^*∗*^	^*∗*^	^*∗*^	^*∗*^	^*∗*^	^*∗*^
(B)	^*∗*^	^*∗*^	^*∗*^	ns	ns	^*∗*^	ns	^*∗*^	ns	^*∗*^	^*∗*^	^*∗*^
(A^*∗*^B)	^*∗*^	^*∗*^	ns	^*∗*^	^*∗*^	ns	ns	^*∗*^	ns	^*∗*^	^*∗*^	ns
C.V. (%)	9.24	7.86	6.35	12.4	10.9	4.19	23.6	4.87	3.74	6.75	5.64	4.54

In a column, the different superscripts indicate significant differences at 5% (*P* < 0.05, ^*∗*^) and without a superscript indicates no significant difference according to Duncan's post hoc test at 5% level. Values are means of four replications.

**Table 4:** Effects of different levels of a mixture of four strains of *Rhodopseudomonas palustris* in spent rice straw and chemical fertilizer on nutrient uptake in parts of sesame grown in dyked alluvial soil under the greenhouse condition.

Factor	Nitrogen uptake			Phosphorus uptake			Potassium uptake		
	(g pot^−1^)								
	Stem and leaf	Shell	Seed	Stem and leaf	Shell	Seed	Stem and leaf	Shell	Seed
Biofertilizer level (A) (t ha^−1^)									
0	0.010^c^	0.009^d^	0.083^c^	0.035^c^	0.027^d^	0.072^c^	0.322^b^	0.092^c^	0.012^c^
3	0.014^b^	0.011^c^	0.107^b^	0.045^b^	0.032^c^	0.087^b^	0.423^a^	0.117^b^	0.017^b^
4	0.015^b^	0.013^b^	0.122^a^	0.045^b^	0.039^a^	0.099^a^	0.418^a^	0.122^b^	0.020^a^
5	0.018^a^	0.014^a^	0.122^a^	0.051^a^	0.035^b^	0.098^a^	0.455^a^	0.168^a^	0.020^a^
Nitrogen and phosphorus fertilizer level (B) (%)									
100	0.018^a^	0.015^a^	0.141^a^	0.052^a^	0.039^a^	0.111^a^	0.466^a^	0.152^a^	0.022^a^
75	0.013^b^	0.011^b^	0.101^b^	0.044^b^	0.031^b^	0.084^b^	0.416^b^	0.120^b^	0.016^b^
50	0.012^b^	0.009^c^	0.085^c^	0.036^c^	0.029^b^	0.072^c^	0.331^c^	0.101^c^	0.014^c^
Significant differences									
(A)	^*∗*^	^*∗*^	^*∗*^	^*∗*^	^*∗*^	^*∗*^	^*∗*^	^*∗*^	^*∗*^
(B)	^*∗*^	^*∗*^	^*∗*^	^*∗*^	^*∗*^	^*∗*^	^*∗*^	^*∗*^	^*∗*^
(A^*∗*^B)	^*∗*^	^*∗*^	ns	^*∗*^	^*∗*^	ns	^*∗*^	^*∗*^	ns
C.V. (%)	12.7	9.89	5.05	12.3	1.08	5.06	8.39	2.38	1.34

In a column, the different superscripts indicate significant differences according to Duncan's post hoc test at 5% level (*P* < 0.05, ^*∗*^). Values are means of four replications.

**Table 5:** Effects of different levels of a mixture of four strains of *Rhodopseudomonas palustris* in spent rice straw and chemical fertilizer on yield components and yield of sesame grown in dyked alluvial soil under the greenhouse condition.

Factor	Yield components									Yield (g·pot^−1^)
	Length of capsule (cm)	Diameter of capsule (cm)	Number of capsules plant^−1^(fruit)	Number of fulfil capsules plant^−1^(fruit)	Percentage of fulfil capsules plant^−1^(%)	Number of seeds capsule^−1^(seed)	Number of fulfil seeds capsule^−1^(seed)	Percentage of fulfil seeds capsule^−1^(%)	Weight of 1,000 seeds (g)	
Biofertilizer level (A) (t·ha^−1^)										
0	2.40^c^	1.08	12.6^c^	11.9^c^	96.1	119.0^b^	112.2^c^	94.4^b^	2.44	10.3^c^
3	2.45^bc^	1.10	15.3^b^	14.8^b^	96.2	124.6^ab^	118.3^b^	95.0^b^	2.47	12.3^b^
4	2.56^a^	1.12	16.7^a^	15.6^a^	94.4	124.1^ab^	123.9^a^	99.7^a^	2.44	14.5^a^
5	2.53^ab^	1.15	17.1^a^	15.9^a^	93.4	125.7^a^	123.1^a^	97.8^a^	2.44	14.3^a^
Nitrogen and phosphorus fertilizer level (B) (%)										
100	2.66^a^	1.19^a^	18.2^a^	17.4^a^	95.8	127.6^a^	126.8^a^	99.3^a^	2.47	15.9^a^
75	2.44^b^	1.10^b^	15.0^b^	13.8^b^	93.2	128.6^a^	123.8^b^	96.3^b^	2.45	12.1^b^
50	2.35^c^	1.05^b^	13.0^c^	12.5^c^	96.2	113.9^b^	107.6^c^	94.7^b^	2.42	10.5^c^
Significant differences										
(A)	^*∗*^	ns	^*∗*^	^*∗*^	ns	ns	^*∗*^	^*∗*^	ns	^*∗*^
(B)	^*∗*^	^*∗*^	^*∗*^	^*∗*^	ns	^*∗*^	^*∗*^	^*∗*^	ns	^*∗*^
(A^*∗*^B)	ns	ns	^*∗*^	ns	ns	^*∗*^	^*∗*^	ns	ns	^*∗*^
C.V. (%)	3.82	6.36	6.02	7.33	5.09	5.33	2.50	4.14	3.61	4.57

In a column, the different superscripts indicate significant differences at 5% (*P* < 0.05, ^*∗*^) and without a superscript indicates no significant difference according to Duncan's post hoc test at 5% level. Values are means of four replications.

**Table 6:** Effects of different levels of a mixture of four strains *Rhodopseudomonas palustris* in spent rice straw and chemical fertilizer on dyked alluvial soil fertility under the greenhouse condition after sesame harvest.

Factor	pH_H2O_(1 : 2.5)	pH_KCl_(1 : 2.5)	Total nitrogen (%·N)	Available ammonium (mg·kg^−1^)	Total phosphorus (%·P)	Soluble phosphorus (mg·P·kg^−1^)	Insoluble calcium-phosphorus(mg·P·kg^−1^)	Insoluble aluminum-phosphorus(mg·P·kg^−1^)	Insoluble iron-phosphorus(mg·P·kg^−1^)	Organic matter (%C)	Exchangeable potassium (meq 100 g^−1^)	Log PNSB (MPN g^−1^DSW)
Biofertilizer level (A) (t·ha^−1^)												
0	6.09^c^	5.12^d^	0.185^c^	98.2^c^	0.031^c^	28.8^d^	146.1^a^	72.9^a^	329.4^a^	1.92^c^	0.353^c^	1.94^c^
3	6.28^b^	5.43^c^	0.213^b^	87.8^d^	0.039^b^	34.9^c^	123.4^b^	62.9^b^	317.4^b^	2.19^b^	0.445^b^	6.14^b^
4	6.35^b^	5.56^b^	0.232^a^	128.3^b^	0.039^b^	37.5^b^	127.1^b^	56.3^c^	316.3^b^	2.25^b^	0.479^b^	6.22^b^
5	6.48^a^	5.73^a^	0.232^a^	136.5^a^	0.041^a^	43.6^a^	124.8^b^	51.8^d^	317.3^b^	2.67^a^	0.543^a^	6.92^a^
Nitrogen and phosphorus fertilizer level (B) (%)												
100	6.30	5.45	0.222	126.2^a^	0.038	39.1^a^	137.5^a^	63.8^a^	324.9^a^	2.33	0.443	5.26
75	6.28	5.45	0.211	108.3^b^	0.037	34.5^b^	132.1^b^	61.3^ab^	323.1^a^	2.25	0.476	5.31
50	6.32	5.47	0.213	103.7^c^	0.037	35.2^b^	121.6^c^	57.7^b^	312.3^b^	2.28	0.446	5.35
Significant differences												
(A)	^*∗*^	^*∗*^	^*∗*^	^*∗*^	^*∗*^	^*∗*^	^*∗*^	^*∗*^	^*∗*^	^*∗*^	^*∗*^	^*∗*^
(B)	ns	ns	ns	^*∗*^	ns	^*∗*^	^*∗*^	^*∗*^	^*∗*^	ns	ns	ns
(A^*∗*^B)	^*∗*^	ns	ns	^*∗*^	ns	^*∗*^	ns	ns	ns	^*∗*^	ns	^*∗*^
C.V. (%)	1.88	2.24	9.29	4.04	5.18	10.6	4.93	8.75	2.02	9.23	11.7	3.18

In a column, the different superscripts indicate significant differences at 5% (*P* < 0.05, ^*∗*^) and without a superscript indicates no significant difference according to Duncan's post hoc test at 5% level. Values are means of four replications. DSW: dry soil weight.

## Data Availability

The data presented in this article are available from the corresponding author upon request.
